# Hereditary Inclusion Body Myopathy (HIBM2)

**DOI:** 10.4137/grsb.s2594

**Published:** 2009-10-21

**Authors:** Chris M. Jay, Nick Levonyak, Gregory Nemunaitis, Phillip B. Maples, John Nemunaitis

**Affiliations:** 1 Gradalis, Inc; 2 Mary Crowley Cancer Research Centers, Dallas, TX, USA; 3 MetroHealth Medical Center, Cleveland, OH, USA; 4 Texas Oncology, PA, USA; 5 Baylor Sammons Cancer Center, Dallas, TX, USA

**Keywords:** HIBM2, GNE, sialic acid, myopathy, neuromuscular

## Abstract

Hereditary inclusion body myopathy type 2 (HIBM2) is a myopathy characterized by progressive muscle weakness with early adult onset. The disease is the result of a recessive mutation in the Glucosamine (UDP-N-acetyl)-2-epimerase/N-acetylmannosamine kinase gene (GNE), which results in reduced enzyme function and sialic acid levels. A majority of individuals with HIBM2 are from Iranian-Jewish or Japanese decent, but isolated cases have been identified world wide. This article reviews the diagnostic criteria for HIBM2. Current research with a highlight on the biology of the disease and the role of GNE in the sialic acid pathway are assessed. Finally, therapeutic investigations and animal models are discussed with a focus on future studies to better understand the pathology of Hereditary Inclusion Body Myopathy and move therapeutic agents towards clinical trials.

## Introduction to HIBM2

HIBM2 is a myopathy that was first recognized in individuals of Iranian-Jewish decent that is clinically manifest by progressive muscle weakness and genetically transmitted in an autosomal-recessive manner. There are a number of myopathies which cause progressive muscle weakness. HIBM2 is unique in that it is clinically characterized by early adult onset, ethnic predisposition, and quadriceps sparing. HIBM2 is histologically characterized by atrophic muscle fibers with rimmed vacuoles and filamentous inclusions in the absence of inflammatory cells.^1^–^3^

Only 220 (http://www.ncbi.nlm.nih.gov/bookshelf/br.fcgi?book=gene&part=ibm) cases of HIBM2 have been reported worldwide, although more cases may be undiagnosed. The vast majority are from Iranian-Jewish decent. In the Iranian-Jewish population the frequency is greater than 1 in every 1500 individuals. This syndrome has also been described in Japan where the disorder is often referred to as Distal Myopathy with Rimmed Vacuoles (DMRV).^4^

Patients of American, Afghanistan, Iraqui-Kurdish, Irish, German, Mexican, African-American, possibly Egyptian communities, and Italian descents have also been identified.^2^,^5^,^6^

HIBM2 is an autosomal recessive disorder and, therefore, an affected allele must be inherited from both parents in order for a child to phenotypically develop the syndrome. These mutated genes (either homozygous or heterozygous mutations) are then expressed as the hypofunctional enzyme, Glucosamine (UDP-N-acetyl)-2-epimerase/N-acetylmannosamine kinase (GNE), which catalyzes the first two reactions in the production of sialic acid. HIBM-associated *GNE* mutations have been shown to reduce sialic acid production.^7^,^8^ The pathologic mechanism for muscle degeneration in HIBM2 remains unknown but evidence suggests, that proper folding, stabilization, and function of skeletal muscle glycoproteins require muscle fiber sialylation.^9^–^12^ *GNE* mutations resulting in hyposialyation of muscle glycoproteins appear to contribute to myofibrillar degeneration and loss of normal muscle function.^9^,^11^,^13^–^16^

The most prevalent mutation in HIBM2 patients of Middle Eastern descent is the missense mutation, M712T, located on exon 12 of the *GNE* gene.^17^,^18^ However, novel mutations (such as the D176V [exon 3] and V572L [exon 9] mutation which is more common in Japanese patients) have also been discovered in both the epimerase and kinase domains of the *GNE* gene in HIBM2 patients of other ethnic backgrounds.^2^,^4^–^6^

This review will summarize diagnostic, molecular, and therapeutic results related to HIBM2.

## Diagnosis

The most common criteria used for identifying HIBM2 are as follows: 1) distal skeletal muscle weakness beginning in the lower legs; 2) selective sparing of the quadriceps; 3) onset in the third to forth decade of life; 4) a familial history of muscle wasting disorder is not obligatory for the diagnosis; 5) minimal to no elevation in serum creatine kinase (CK); 6) the histologic presence of rimmed vacuoles within muscle fibers in the absence of inflammatory cells; and 7) tubofilamentous inclusions in muscle by electron microscopy. Exceptions have been described; however, they are generally applicable to less than 10% of patients.^18^

One unscrutinized criteria for diagnosis of HIBM2 is the onset in early adulthood. Generally, the disorder becomes apparent anywhere from 20 to 40 years of age. After 2–3 decades, almost complete muscle function is lost in the patient with sparing of the facial, extraocular, bulbar, intercostal and diaphragm muscles.^19^ The progressive deterioration of the axial muscles will often leave the patient wheel-chair bound and unable to perform the basic functions of everyday life.

## Molecular Biology

Underproduction of skeletal muscle sialic acid related to dysfunctional GNE gene product is the hallmark of HIBM2.^5^,^9^,^13^,^18^,^20^ Sialic acid is the only sugar that bears a net negative charge.^21^ This charged sugar provides the terminal carbohydrate on a variety of N-linked and O-linked glycoproteins that mediate cell-cell and protein-protein interactions. Thus, the role as cell surface macromolecules may not be easily mimicked by other biologically available oligosaccharide moieties. More than 40 different sialic acid compounds have been identified with various biological functions. Sialic acids can be found as part of cell surface glycoproteins, glycolipids, gangliosides, and polysaccharides. The sialic acid modifications of cell surface glycoproteins are crucial for cell adhesion and signal transduction and may result in muscle fiber degeneration.^22^,^23^

**Figure 1.**
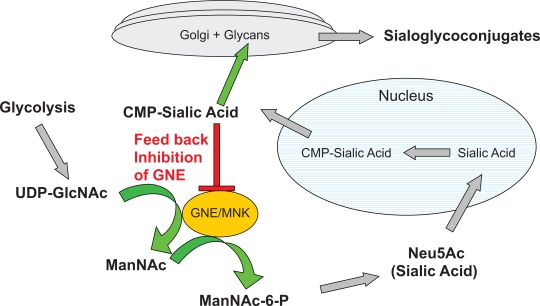
Sialic acid pathway. GNE/MNK is the rate limiting step in the pathway. The downstream product, CMP-sialic acid regulates the activity of GNE by allosteric inhibition.

UDP-GlcNAc 2-epimerase activity is rate-limiting for the biosynthesis of sialic acid. The activity of the enzyme can be controlled by allosteric regulation of the downstream product, CMP-sialic acid (Fig. 1).^24^ In cardiac muscle, removal of sialic acid produces a large specific increase in sarcolemmal calcium permeability without perturbation of potassium permeability.^12^,^25^,^26^ Sialic acid accounts for a component of negatively charged sites which, with other acidic mucopolysaccharides, contributes to cationic binding at the surface of the cell. Calcium bound at the surface seems to be of importance in the excitation-contraction (EC) coupling sequence whether as a source of “trigger” calcium for the sarcotubular system or as a direct activator of the myofilaments. The bound Ca^++^ appears to be in rapid equilibrium with free Ca^++^ in the vascular and interstitial spaces and is the probable immediate source of the Ca^++^ that crosses the sarcolemma. The integrity of the glycocalyx appears to be necessary in the prevention of uncontrolled entry of Ca^++^ into the cell.^12^,^27^

In skeletal muscle, most transmembrane proteins, including voltage-gated sodium channels, are post-translationally modified. Channels are heavily glycosylated, with up to 40% of the molecular weight of the functional sodium channel comprised of sugar molecules. It has been shown that as the sugar residues are removed, sodium channel activity is changed such that a greater depolarization is needed to activate the channel. Thus, channels with lowered glycosylation levels will be less active at a given membrane potential, and the cell likely will be hypo-excitable.^28^

Loss of GNE activity in HIBM2 is thought to impair sialic acid production and interfere with proper sialylation of glycoconjugates. Sialylation of the voltage-gated sodium channels is critical to maintain proper gating of sodium for effective initiation and propagation of action potentials in nerve and muscle.^29^–^35^ Voltage-gated sodium channels are complex membrane proteins composed of an α subunit and one or more smaller β subunits.^36^–^38^ Estimates indicate that 15%–40% of the total sodium channel molecular weight is carbohydrate.^39^–^41^ Approximately 40%–45% of the added carbohydrate residues are sialic acid.^40^,^41^ The terminal sialic acid residues are attached to α and β subunits and in the absence of sialic acid the channel gating is in the depolarized position.^42^–^46^ The lack of effective action potential transmission results in the failure of nerve and muscle activation.

Voltage-gated sodium channels (Na_v_) are responsible for initiation and propagation of nerve, skeletal muscle, and cardiac action potentials. Na_v_ are composed of a pore-forming α-subunit and often one to several modulating β-subunits. Previous work showed that terminal sialic acid residues are attached to α-subunits and affect channel gating.^28^ Under conditions of reduced sialylation, the β1-induced gating effect is eliminated. Consistent with this, mutation of β1 N-glycosylation sites abolish all effects of β1 on channel gating. Data also suggest an interaction between the cis effect of α-sialic acids and the trans effect of β1 sialic acids on channel gating. β1 sialic acids have no effect on gating of the heavily glycosylated skeletal muscle α-subunit. However, when glycosylation of the skeletal muscle α-subunit was reduced through chimeragenesis such that α-sialic acids did not impact gating, β1 sialic acids caused a significant hyperpolarizing shift in channel gating. Together, the data indicate that β1 N-linked sialic acids can modulate Na_v_ gating through an apparent saturating electrostatic mechanism.^46^

Previous studies have shown that the subunits of the Na^+^ channel are modified by glycosylation and the β1, β2, brain, and muscle α-subunits are heavily glycosylated, with up to 40% (eel electroplax α-subunit)^47^ of the mass being carbohydrate. In contrast, the cardiac subunit is only 5% sugar by weight.^48^ Sialic acid is a prominent component of the N-linked carbohydrate of the Na^+^ channel. The addition of such a highly charged carbohydrate has predictable effects on the voltage dependence of gating through alteration of the surface charge of the channel protein. Neuraminidase treatment to remove cell surface sialic acid from skeletal muscle channels produced a depolarizing shift of steady-state inactivation.^43^ Local surface charge is also significantly influenced by charged amino acid residues which stud the outer mouth of the pore, although the predominant effects in this case are on permeation rather than gating.^49^,^50^

Co-translational glycosylation is essential for the maintenance of cell surface expression of the Na^+^ channel in neurons and Schwann cells.^51^,^52^ Inhibition of glycosylation by tunicamycin reversibly decreases the number of STX binding sites on neuroblastoma cells.^53^ Tunicamycin also inhibits palmitation, sulphation, and disulphide attachment of the β2 subunit, preventing the assembly of functional Na^+^ channels.^39^

Studies of HIBM2 patients reveal mutations in the GNE gene associated with glycosylation deficiencies which may lead to defective muscle function. Reduced GNE activity is thought to impair sialic acid production and interfere with proper sialylation of glycoconjugates.^12^ The reactivities to lectins are also variable in some myofibers, suggesting that hyposialylation in muscles may contribute to the focal accumulations of autophagic vacuoles and/or amyloid deposits in affected muscle tissue. Although sialic acid dysregulation is likely primary to disease pathogenesis, recent assessments of myoblast cellular sialylation patterns,^14^,^15^ suggest the possible role of other GNE-related contributing mechanisms.^54^,^55^ Eisenberg et al looked at the role of GNE gene and other neighboring genes, such as, clathrin light chainA (CLTA)^56^ which is a regulatory element in clathrin gene function, known to be involved in several pathways of lysosomal proteolysis, and, reversion-inducing cysteine-rich protein with Kazal motifs (RECK)^57^ which is a membrane-anchored glycoprotein with transformation suppressor activity both located close to the GNE gene.^58^ Sequencing of the coding regions of these genes, and LOC64148 and FLJ21343, which also could not be excluded as possible functional candidate genes in HIBM, revealed no disease causing alleles in any of these genes in HIBM families. Although RECK and CLTA are located close to GNE on chromosome 9p12, there was no evidence that these genes contributed to HIBM2.

Neprilysin, also known as neutral endopeptidase (NEP), CD10, and common acute lymphoblastic leukemia antigen (CALLA), is a zinc-dependent metalloprotease enzyme that degrades a number of small secreted peptides, most notably the amyloid beta peptide whose abnormal misfolding and aggregation in neural tissue has been implicated as a cause of Alzheimer’s disease. One study found that Neprilysin participates in skeletal muscle regeneration and is accumulated in abnormal muscle fibres of inclusion body myositis.^59^ NEP may play an important role during muscle cell differentiation, possibly through regulation, either directly or indirectly, of the insulin-like growth factor I-driven myogenic programme. However, IGF-1 does not appear to be a contributing factor in HIBM2.^59^

Although sialic acid dysregulation is key to disease pathogenesis, recent assessment of myoblast cellular sialylation in patients suggests the role of other GNE related mechanisms including that of α-actinin 1. α-Actinin 1 is one in a group of four α-actinins and they all play key roles in cell-cell contact sites as well as stress fiber dense regions. There is a direct kinetic relationship between the GNE gene and α-actinin 1 which could also lead to muscle deterioration along with sialic acid. Calcium has been shown to have a positive effect on α-actinin.^60^

To investigate the role of mutated GNE enzyme, tissue derived cell cultures from biopsies carrying either kinase or epimerase mutations were created.^14^,^15^ All mutations in the GNE gene caused a reduction in epimerase activity but only the homozygous epimerase mutation actually showed a reduction in sialic acid. However, Penner et al found that recombinant expressed GNE mutants containing either epimerase or kinase mutations had reduced epimerase enzyme activity, with the exception of the most common mutation, M712T, which had normal epimerase activity, but reduced kinase activity.^8^ Therefore, the mutations in the two domains do not have the same effect on enzyme activity or sialylation of muscle cells. The sialic acid modifications of cell surface glycoproteins are crucial for cell adhesion and signal transduction and may result in muscle fiber degeneration.^22^,^23^

Although GNE mutations are widely accepted as the root cause of HIBM2, there are some discussions that GNE plays a role in functions beyond sialic acid synthesis. Upon nocodazole treatment to inhibit intracellular trafficking, GNE was redistributed from the Golgi to the cytoplasm. This suggests that GNE may play a role as a nucleocytoplasmic shuttling protein.^61^ Salama et al demonstrated in vitro that myoblasts and lymphoblastoid cell lines derived from HIBM2 patients containing the M712T mutation had reduced epimerase activities, but did not display reduced membrane bound sialic acid. However, clinical samples from HIBM2 patients demonstrated clear reduction in sialic acid levels from muscle tissues.^9^,^11^ Savelkoul may have described it best that HIBM2 defects in sialylation may appear gradually and tissues such as muscle, which normally express low levels of GNE protein, are more sensitive to disruptions in the GNE enzyme and hence reduced sialic acid expression.^62^

GNE is ubiquitously expressed in all tissues, although at relatively different levels in each specific tissue. GNE is expressed at high levels in the liver, and by comparison at relatively low levels in skeletal muscle. Krause et al found that GNE protein is expressed in skeletal muscle at equal levels in HIBM patients and normal control subjects. Furthermore, immunofluorescence detection of GNE did not reveal any mislocalization of GNE in skeletal muscle of HIBM patients. Thus, most in the field conclude that impaired GNE function, not lack of expression, is the key pathogenic factor in HIBM.^63^ In fact, Penner et al characterized several different GNE mutations and demonstrated that unique mutations altered activity of GNE enzyme to varying degrees of severity, as assessed by downstream enzyme kinetics of ManNAc phosphorylation using a radiolabeled phosphate assay.^8^ Interestingly, all mutations did retain a minimal amount of activity relative to the wild type GNE enzyme.

## Role of GNE in the Sialic Acid Pathway

The GNE gene encodes the bifunctional enzyme UDP-GlcNAc 2 Epimerase/ManNAc Kinase (GNE/MNK). This enzyme is the rate-limiting step which catalyzes two sequential reactions committed towards sialic acid biosynthesis (Fig. 1). The product of the pathway, cytidine monophosphate-N-Acetylneuraminic Acid (CMP-sialic acid), binds to the allosteric site of the GNE enzyme and inhibits the rate limiting epimerase reaction.

The sialic pathway begins with the glycolysis cycle, which produces UDP-GlcNAc. The GNE/MNK bifunctional enzyme converts the UDP-GlcNAc into ManNAc and then ManNAc-6-P. This sugar is then converted into sialic acid via NeuAc-9-P synthase/phosphatase and transported into the nucleus, where CMP-sialic acid synthase adds CMP to Neu5Ac.

CMP-sialic acid leaves the nucleus and is transported to the golgi where sialyltransferase binds sialic acid and glycans to create sialoglycoconjugates. CMP is released during this step and recycled back to the nucleus. Any excess CMP-sialic acid in the cytosol will bind to the regulatory domain of GNE and block further conversion of UDP-GlcNAc into ManNAc. Therefore, the downstream product CMP-sialic acid is able to bind to the auto-regulatory domain of GNE and prevent the overexpression of sialic acid and sialoglycoconjugates.

## Therapeutic Investigation

Currently there is no known effective therapy for the treatment of HIBM2. While other myopathies such as polymyositis and dermatomyositis respond at least partially to corticosteroids, plasmaphoresis/filtration, or other immunosuppressive therapies, there is no or limited evidence of efficacy with these approaches in HIBM2.^64^,^65^ Review of the literature failed to identify clinical trials using steroids and plasmaphoresis for the treatment of persons with HIBM2, however the use of intravenous immunoglobulins (IVIG) has been studied. In a double-blind, placebo-controlled trial involving 19 individuals with HIBM2 treated with IVIG conducted in 1997, results suggested that this treatment may have a very short term affect on some patients.^66^ In a more recent study conducted in 2007, four HIBM patients were treated with IVIG and each showed improved muscle function throughout the study.^67^ However, while these patients showed improved muscle function, there was no evidence to suggest that glycoprotein sialylation was positively effect by the immune globulins. It was postulated that the IVIG would supply sialic acid (IVIG being sialic acid rich). No direct evidence was given to support this. It was not thought that the IVIG was mediating some immune mediated issue. There was no functional benefit observed when the IVIG was combined with prednisone in another study involving 36 patients.^68^

Sialic Acid, ManNAC, or NeuAc replacements, which bypass the defective epimerase and/or kinase related to the GNE mutation, are currently being explored in murine and in vitro experiments and preliminary results show potential improvement in GNE mutated fibroblast function.^7^,^22^,^69^

A study conducted in early 2007 provided evidence that a GNE knockout mouse which expressed the human mutated *GNE* gene with D176V mutation (Gne^(−/−)^ hGNED176V-Tg) developed similar features to human HIBM2 patients. The mice began with just the addition of a Neo cassette that replaced 1.4 kb upstream of exon 3, through 1.4 kb downstream of exon 3, thereby deleting exon 3. However, using this procedure only wild type (WT) and GNE^(+/−)^ mice survived. However, when GNE^(+/−)^ mice were crossed with a transgenic mouse that expressed the human mutated GNE with D176V (which is one of the most common mutations), 9% of the offspring were of the desired Gne^(−/−)^ hGNED176V-Tg.^70^

The sialic acid levels of both the Gne^(−/−)^ hGNED176V-Tg mice and the WT mice were measured using high-performance liquid chromatography. Sialic acid levels were measured in the liver, spleen, brain, kidney, muscle, and heart. For WT mice, levels were highest in the liver, spleen, and brain, but the sialic acid levels of the Gne^(−/−)^ hGNED176V-Tg mice were much lower. In fact, in the liver and kidney, WT mice showed more than five times the amount of sialic acid. Gne^(−/−)^ hGNED176V-Tg had a third less sialic acid than WT species in the spleen. Overall, it is clear that the Gne^(−/−)^ hGNED176V-Tg have an overall decrease in sialic acid.^70^

Progression of the disease also seemed to be noticeably similar in the Gne^(−/−)^ hGNED176V-Tg to human HIBM2 patients. At birth, the Gne^(−/−)^ hGNED176V-Tg mice were not distinguishable from the other mice, but after 30 weeks of age these mice weighed less than the WT mice. Thirty weeks in the lifetime of a mouse is comparable to the second to third decade onset in human patients. When 12 of the Gne^(−/−)^ hGNED176V-Tg mice died, 5 (41%) mice had rimmed vacuoles (RVs) in their skeletal muscles which is a key histological characteristic of HIBM2.^70^

Given this finding, characteristic pathological features were then tracked in new litters of Gne^(−/−)^ hGNED176V-Tg mice. Again, before 30 weeks of age, there was almost no difference observed between the Gne^(−/−)^ hGNED176V-Tg and their littermates. By 40 weeks of age, fibers begin to appear atrophic and RVs are spotted in scattered fibers. On occasion, inclusion bodies were found in the fibers with or without RVs. Similar to humans, these RVs have been intensely stained with acid phosphatase, indicating that autophagic process is activated.^70^

From these studies, it is quite clear that the Gne^(−/−)^ hGNED176V-Tg is the closest model to date that resembles human HIBM2 patients both histologically and pathologically. However, there are several differences in the D176V-Tg mouse compared with HIBM2 patients. The mice had a high GNE D176V-Tg mRNA expression in the muscle, possibly due to the promoter used in the study. Also, the quadriceps were preferentially involved in the mice, whereas HIBM2 patients are characterized by quadriceps sparing until late into the disease progression. Finally, some of the GNE D176V-Tg mice died sooner than their litter-mates for unknown reasons. Studies conducted before had failed to produce a mouse that could actually be tested for treatment because the animals were embryonic lethal.^34^

A research article published in summer 2007 outlines the creation of a Gne^M712T/M712T^ knockin mouse that was then actually treated using ManNAc feeding as an exploratory therapeutic measure. The mouse was created using a murine targeting vector for homologous recombination in C57BL/6J embryonic stem cells which included the M712T mutation. The neomycin phosphotransferase and thymidine kinase genes were each respectively introduced into the vector as positive and negative selection markers. This entire vector was sequence verified.^71^

As with the previous study, there were non-HIBM2 phenotypes present in the M712T mouse model, along with indicators that these mice showed similar pathology to human HIBM2 patients. Untreated Gne^M712T/M712T^ pups died by day 3 due to severe glomerular proteinuria. High-magnification examination of the Gne^M712T/M712T^ kidneys indicated that red blood cell infiltrates in the proximal and distal convoluted tubules and collecting ducts were present in the mutated mice. This renal phenotype is not present in HIBM2 patients.

The second portion of this M712T mouse study was to administer ManNAc to drinking water of pregnant females during gestation and early postnatal nursing of mutant mice. ManNAc is situated in the sialic acid pathway after the regulated rate-limiting GNE step so its metabolism is not subject to feedback inhibition. Given at a concentration of 1 mg/mL, the ManNAc produced no surviving homozygous Gne ^M712T/M712T^ mice out of 51 total offspring. However, when the concentration was increased to 5 mg/ml, 12 Gne^M712T/M712T^ mice (12%) survived beyond P3 out of a total of 102. Of the pups that survived beyond day 3,17% survived beyond weaning (day 21), when ManNAc treatment was stopped. These surviving mice continued to grow until 3.5 months, but remained smaller than their littermates. No side affects were observed due to the treatment and upon ManNAc treatment. There were less cystic tubular dilations in the cortex and medulla supporting histological improvement in these mice as well. Biochemical analysis also demonstrated improvement in GNE protein expression, PSA-NCAM, and podocalyxin. These results support the evaluation of ManNAc therapy for the treatment of HIBM2.^71^

In another study, GNE gene replacement demonstrated the safety of GNE-lipoplexes administered to normal mice.^72^ A GNE-wt-DNA vector using human GNE cDNA and the pUMVC3 expression vector was constructed and placed in an extensively characterized cationic liposome.^73^,^74^ This vector produced high levels of recombinant GNE protein and subsequent sialic acid in transfected CHO-Lec3 (GNE deficient cell line) cells that produced low levels of sialic acid.^73^ The lipoplexes were injected intramuscularly (IM) or intravenously (IV) into BALB/c mice. Single IM injections of the GNE-lipoplex at 40 μg DNA did not produce overt toxicity or deaths, indicating that the maximum tolerated dose for IM injection was ≥40 μg DNA. In fact, mice administered with either 10 μg or 40 μg GNE-lipoplex survived without demonstrating overt signs of toxicity for the observation period of 2 weeks. These findings were supported by 100% survival in IM injected mice and the lack of hematology, blood chemistry, or histological abnormalities since the maximum tested dose was 40 μg. Single intravenous (IV) injections of GNE-lipoplex was lethal in 33% of animals at 100 μg dose, while mice injected at 40 μg exhibited no toxicity or pathology. This indicates that the maximum tolerated IV dose is somewhere between 40–100 μg.

Real-time RT-qPCR analysis demonstrated recombinant human GNE mRNA expression in all muscle tissues that received IM injection of 40 μg GNE DNA-lipoplex at 2 weeks post-injection. These results indicate that GNE-lipoplex gene transfer is safe and can produce durable transgene expression in treated muscles.^72^

Another investigation provided evidence that sialylation is essential for the early development of mice and that the inactivation of UDP-GlcNAc 2-epimerase via gene therapy results in early embryonic lethality in mice.^75^ This early lethality in the GNE deficient mice is most likely explained by the loss of protection from proteolytic processes or by disturbed cell-cell adhesion because almost all cell adhesion molecules are sialylated glycoproteins.^75^ This is evidence that functional GNE enzyme and sialic acid are both necessary for mice and humans.

The same investigators then looked at how the GNE deficient mice differed from wild-type species. Previous evidence showed that GNE–deficiency, on average, was lethal at day 8.5 of embryonic development.^76^ Similar to Malicdan, the sialic acid levels of key organs were taken to differentiate between the sialylation of GNE-deficient and wild-type species.^76^ Again, wild type mice had a greater expression of sialic acid in every organ except the kidney. Overall, in GNE-deficient mice, there was a 25% reduction in membrane-bound sialic acids.^76^ Studies involving HIBM2 patients demonstrated reduced sialylation on neural cell adhesion molecule (NCAM)^11^ and α-dystroglycan^9^ in muscle tissues.

## Discussion

Until recently, little hope for recovery of muscle function has existed for patients with HIBM2. However with the advent of molecular biology and characterization of etiologic pathology, particularly involving the GNE gene; GNE gene replacement may provide an opportunity to better characterize the pathology of this rare disease and to provide possible therapeutic value to patients. Continued development of relevant animal models may facilitate such studies. Clinical investigation of intravenous administration of GNE plasmid—lipoplex product is a fruitful area of research. The rarity of this syndrome and the lack of existing therapeutics allow for the pursuit of an orphan drug development program if safety and evidence of benefit to afflicted patients are demonstrated. Other delivery vehicles may also be considered and tested to enhance activity. Furthermore, once functional GNE expression is augmented in patients (and muscle function improved), it may provide further insight into involvement of other downstream effector pathways. Exploring the expression of molecular signaling patterns of skeletal muscles in parents of early stage and late stage HIBM2 patients to better understand the mechanism of the onset of this disease may enable early preventive approaches to be implemented.
